# Electronic Structures of Clusters of Hydrogen Vacancies on Graphene

**DOI:** 10.1038/srep15310

**Published:** 2015-10-15

**Authors:** Bi-Ru Wu, Chih-Kai Yang

**Affiliations:** 1 Department of Natural science, Center for General Education, Chang Gung University, Kueishan 333, Taiwan, ROC; 2 Graduate Institute of Applied Physics, National Chengchi University, Taipei 11605, Taiwan, ROC

## Abstract

Hydrogen vacancies in graphane are products of incomplete hydrogenation of graphene. The missing H atoms can alter the electronic structure of graphane and therefore tune the electronic, magnetic, and optical properties of the composite. We systematically studied a variety of well-separated clusters of hydrogen vacancies in graphane, including the geometrical shapes of triangles, parallelograms, hexagons, and rectangles, by first-principles density functional calculation. The results indicate that energy levels caused by the missing H are generated in the broad band gap of pure graphane. All triangular clusters of H vacancies are magnetic, the larger the triangle the higher the magnetic moment. The defect levels introduced by the missing H in triangular and parallelogram clusters are spin-polarized and can find application in optical transition. Parallelograms and open-ended rectangles are antiferromagnetic and can be used for nanoscale registration of digital information.

Graphane is the end product of the complete hydrogenation of graphene^1^,^2^,^3^,^4^,^5^,^6^,^7^,^8^,^9^,^10^,^11^,^12^,^13^,^14^,^15^,^16^,^17^,^18^,^19^,^20^. Carbon atoms in graphane are bonded to H atoms alternately from either side of the plane of graphene. Metal-insulator transition^1^,^2^,^3^,^4^ occurs as a result of the process, opening a large band gap^6^,^7^,^8^,^9^,^10^,^11^ for graphane. However, it is possible that the hydrogenation process is not thorough and some H vacancies are left as defects in graphane. H vacancies in graphane are thus defined as C atoms not bonded to H. In a more controllable manner, some H atoms can desorb from one side of graphane as a result of an applied electric field^12^,^13^,^20^, and the desorption process may continue to the extent of half hydrogenation. Patterns of H vacancies in graphane^12^,^13^,^14^,^15^,^19^,^21^,^22^,^23^,^24^ can thus be formed and have attracted considerable interests^3^,^6^,^10^,^11^,^12^,^13^,^14^,^15^,^16^,^17^,^18^,^19^,^20^,^21^,^22^,^23^,^24^,^25^,^26^,^27^,^28^,^29^,^30^,^31^,^32^ as partially hydrogenated graphene or, equivalently, graphane with patches or clusters of H vacancies, can have very different electronic structure from either pristine graphene or graphane, providing almost unlimited ways for designing and fine-tuning electronic circuits based on the two-dimensional composite of C and H. In this article we would like to report investigations by density functional theory (DFT) on some geometric patterns of H vacancies in graphane and their physical properties. The results can be applied to the design of nanoelectronic circuits^9^,^10^,^25^ and may serve as a guide for predicting properties of larger and more complicated patterns of the C-H composites.

H vacancies can be continuously distributed over a vast area and simulated by a large periodic structure. H vacancies can also be confined in a finite area of graphane. Continuous presence of H vacancies can tune the width of band gap of graphane^33^,^34^ and, in the case of a single H-vacancy chain, even turns the defected graphane into a conductor with linear band dispersion^33^ near the Fermi level. Locally distributed H vacancies are more like quantum dots, offering essentially dispersionless defect states in the band gap. We chose a few geometric shapes of H-vacancy dots for calculation based on DFT. These highly symmetric clusters or dots of H vacancies serve as building blocks for more general and complicated patterns. Together with H-vacancy chains, they can be assembled for the design of a large variety of nanoelectronic circuits.

Starting with a cluster of four H vacancies as shown in Fig. 1a, we gradually enlarged the triangular dot to the one containing 121 vacancies in the process. Each equilateral triangle was placed in a large unit cell for DFT calculation to ensure its isolation from the triangles in adjacent cells. Relaxation of all atomic positions was always executed for the purpose of obtaining an optimal configuration with minimum stress before energy bands and other physical properties were calculated. One important result is the formation energy of individual H vacancies (*E*_f_) defined as


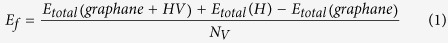


where *E*_total_(graphane + HV) is the total energy of graphane with the dot of H vacancies, *N*_*v*_ the number of H vacancies in the dot, *E*_total_(H) the total energy of *N*_v_ free H atoms, and *E*_total_(graphane) the total energy of pure graphane. Alternate definition of the formation energy can be found in Part II of the Supplementary Information which accompanies this paper. Consistent with previous studies, larger spreading area of H vacancies lowers the energy needed to remove an outlying H atom. As Fig. 2a indicates, formation energy per H vacancy follows a decaying curve from 3.32 eV for the smallest triangle to less than 2.65 eV for those larger than 121 vacancies.

Also in decline as the H-vacancy dot grows larger is the band gap of the graphane containing the triangular dot, as is shown in Fig. 2b. The gap has its largest value at 1.56 eV for the smallest triangular dot and is reduced to less than 0.52 eV for the dot containing 121 vacancies. Figure 3a,b show two examples of band structures for the smallest triangular vacancy dot and another with 36 vacancies. They all reveal essentially dispersionless spin-polarized energy bands in the graphane band gap. The flat bands are mostly made of *p*_z_ orbitals contributed by the C atoms in either vacancy dot, with those corresponding to the majority spin directly below the Fermi level and those of the minority spin above. For the larger dot, as Fig. 3b suggests, more spin-polarized flat bands appear in the valence and conduction bands, reflecting presence of more bare C atoms.

The triangular clusters of H vacancies are thus strongly associated with magnetism. As the fitted curve in Fig. 3c indicates, the total magnetic moment *m* of a triangular dot is practically proportional to the square root of the number of vacancies *N*_v_ the dot contains. The equation for the curve is *m* 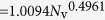 in unit of *μ*_*B*_. It allows design and construction of reliable nanoscale magnets on a two-dimensional circuit. The enhancement of magnetism by the enlargement of vacancy clusters can be visualized by the plots of spin density in Fig. 4, in which the majority spin density greatly outnumbers the minority spin density in each configuration.

Our calculated magnetism for triangular H-vacancy clusters is also consistent with the discussion^35^,^36^ from the perspective of graphene with H adsorption. By considering the bare C atoms as distributed over a bipartite structure^35^ and C atoms of the same sublattice coupled ferromagnetically and those of different sublattices coupled antiferromagnetically, one is able to obtain the same magnetic moment as given in the equation of the last paragraph for each of the triangular vacancy dot in Fig. 1a.

An H-vacancy cluster consisting of two back-to-back equilateral triangular dots has the shape of a parallelogram as shown in Fig. 1b and even number of H vacancies. Its distribution of magnetic moments is drawn in Fig. 5a for parallelograms consisting of the number of C atoms ranging from 18 to 72, in which two tips of any parallelogram have the largest but opposite moments. The other C atoms in the cluster have smaller moments depending on their spatial distances from the tips, with the C atoms at the edges of the cluster tending to shrink more slowly than those in the interior. Total magnetic moment is zero, which also agrees with the prediction based on the bipartite structure with equal number of bare C atoms distributed in two sublattices. The distribution of local moments, however, make the parallelogram an antiferromagnetic unit in which two quantum messages as represented by the opposite spins can be resolved within a few Å. If the two halves of the parallelograms are divided and separated by a single hydrogenated zigzag carbon line, as reported in Ref. 29they are still coupled in antiferromagnetism.

From Fig. 2a, it is clear that for small and midsize (less than 70 C atoms) vacancy clusters, a parallelogram has smaller formation energy than that of a triangle containing the same number of C atoms. The reason is attributed to more exposed C atoms at the edges of the latter. Once the cluster grows large enough with comparable interior C atoms in both cases, the difference in formation energy is practically erased. Band gaps of parallelograms also depend on their sizes, the larger the cluster the narrower the gap. The three band structures drawn in Fig. 5b correspond to the parallelogram dots of 8, 18 and 32 C atoms. Band gap shrinks abruptly from 2.02 eV for the cluster of 8 to 0.98 eV for the 18-C cluster. It rises slightly for the 32-C cluster but then conforms to the declining trend and eventually merges with curve for the triangles in Fig. 2b, consistent with our previous description of the parallelogram as a combination of back-to-back triangles. The energy bands in Fig. 5b are not spin-split despite the sub-nanoscale distribution of spin density inside the cluster.

All hexagonal vacancy clusters are divided into two types that have either zigzag or armchair chains of edge C atoms as depicted in Fig. 1c. Left panel of Fig. 1c is illustration of zigzag-edge hexagonal dots starting from the smallest of 6 C atoms. Right panel of Fig. 1c shows the buildup of armchair-edged hexagonal dots from 12 C atoms. For small hexagonal dots, curves in Fig. 2a indicate that zigzag-edged ones have significantly lower formation energies. Small armchair-edged hexagonal dots need more energy to form because there are fewer nearest-neighbor H atoms in an armchair chain and it takes more energy to remove those H atoms to shape the edge. The difference in formation energy between the two types disappears for large hexagonal vacancy dots.

Band gaps of zigzag-edged hexagonal H-vacancy clusters follow a similar decaying curve shown in Fig. 2b as most other shapes, the larger the cluster the smaller the gap. However, energy gaps of hexagonal clusters tend to be conspicuously higher than those of triangular or parallelogram clusters having comparable number of C atoms. In Fig. 6a,b two typical examples, one with 24 and another with 54 C atoms, are shown with their calculated band structures and charge densities at the highest occupied molecular orbital (HOMO) and lowest unoccupied molecular orbital (LUMO). Energy gap between the HOMO and LUMO drops from 2.56 eV for the cluster of 24 C atoms to 1.73 eV for the cluster of 54 C atoms, both close to the values reported in Ref. 30 for comparable configurations. It is also obvious that charge density for LUMO is more fragmented and has more nodes than that for HOMO in both examples. No magnetism is associated with zigzag-edged hexagonal H-vacancy clusters. The clusters are all made of whole 6-member hexagonal rings and even number of C atoms in the bipartite structure. Local moments and therefore the total magnetic moment disappear.

No simple relation between the band gap and cluster size exists for armchair-edged hexagonal clusters. The smallest one (12 C atoms) has a similar band gap as those of the triangle and parallelogram with comparable number of C atoms. While larger hexagons have higher gaps, there is an exception for the one with 25 C atoms. This particular shape not only has the lowest band gap of only 0.31 eV but carries a magnetic moment of 0.742 *μ*_*B*_ with it. As the energy bands drawn in Fig. 6c reveal, only the 25 C cluster has spin-polarized energy bands, with one dispersionless energy level belonging to the majority spin 0.15 eV below the Fermi level and another to the minority spin also only 0.15 eV above. The 25-C cluster is unique in possessing a magnetic moment in that it is the only armchair-edged cluster in Fig. 1C that simultaneously has broken hexagons and an uneven distribution of C atoms in the two sublattices.

Finally we investigated rectangular H-vacancy dots. Four particular configurations were considered: zigzag-edged clusters with open or closed ends (ZZO or ZZC) and armchair-edged clusters with open or closed ends (ACO or ACC), depending on whether the dot contains only whole hexagons of C atoms. Figure 1d plots the four configurations and Fig. 7a displays the spin density in the two open-ended cases. Although both ACC and ZZC are made of closed hexagons and nonmagnetic, ACO and ZZO are associated with antiferromagnetism with zero total moment for each of the two clusters, in consistent with their more exposed and discontinuous configurations and even distribution of C atoms in the sublattices. It is also obvious that opposite magnetic moments concentrate on the two ends of ACO but spread out along the two edges of ZZO, making the two configurations shape-dependent nanoscale two-pole magnets. Energy bands for the four configurations are drawn in Fig. 7b, confirming the antiferromagnetic properties of the two open-ended structures as the bands corresponding to either spin overlaps. Figure 7b and Fig. 2b also indicate that ACO and ZZO have smaller band gaps than ACC and ZZC, with ZZO’s being the narrowest at 0.82 eV. The fact that both open-ended rectangular H-vacancy dots require more formation energies (Fig. 2a) than their close-ended counterparts is also quite as expected.

The various geometric shapes and sizes of H-vacancy dots we have studied provide a useful map for their application. Band gaps are generally consistent with the calculation of Gao *et al.*^37^, with one exception of armchair-edged hexagonal vacancy dots. Where magnetism is concerned, triangular dots are good candidates for forming molecular magnets. By simply expanding the size equilaterally magnetic moment increases in proportion to the square root of the number of C atoms contained inside. Spin-polarized energy levels near the Fermi level also make optical transitions and sensing^31^ available. It is also possible to manipulate the optical transitions by making regular patterns of triangular dots^32^ segregated far enough from one another. Parallelograms have lower formation energies and are two-pole magnets, in sharp contrast to the triangles. Patterns of alternate presence of triangles, parallelograms, and close-ended rectangles can be used to register digital information in very confined space. A hexagonal cluster tends to have the lowest formation energy but also the largest energy gap in most cases.

In summary, we have presented electronic structures and properties of a variety of H-vacancy clusters in graphane according to their geometric shapes and sizes. These vacancy dots, along with vacancy chains and ribbons, are useful building blocks for further research on related physics based on the platform of graphene. They can also be applied to the design of graphene-based microelectronic circuits.

## Methods

We performed spin-polarized density-functional calculations using the Vienna *ab initio* simulation package (VASP)^38^,^39^. For exchange-correlation functional, the version of Perdew, Burke and Ernzerhof^40^ was adopted. The electron-ion interaction was represented by the projector-augmented wave potential. Cutoff energy for the expansion of wave functions and potentials in the plane-wave basis were chosen to be 500 eV. Complete relaxation of the combined structure including lattice constants was executed with a 9 × 9 × 1 sampling of the first Brillouin zone. In our calculations, vacuum space in the supercell was allocated by setting a height of 15 Å perpendicular to the graphane plane in the cell, which proved large enough to minimize artificial interactions between supercells.

We have also adopted large unit cells in the calculation in order to accommodate various distributions of vacancy dots. A large unit cell containing 288 C atoms, for example, has been used for calculations involving large vacancy dots and for the testing of eliminating inter-dot interaction. All energy bands shown in the figures of this article are from calculations based on the unit cell of 128 C atoms.

## Additional Information

**How to cite this article**: Wu, B.-R. and Yang, C.-K. Electronic Structures of Clusters of Hydrogen Vacancies on Graphene. *Sci. Rep.* **5**, 15310; doi: 10.1038/srep15310 (2015).

## Supplementary Material

Supplementary Information

## Figures and Tables

**Figure 1:**
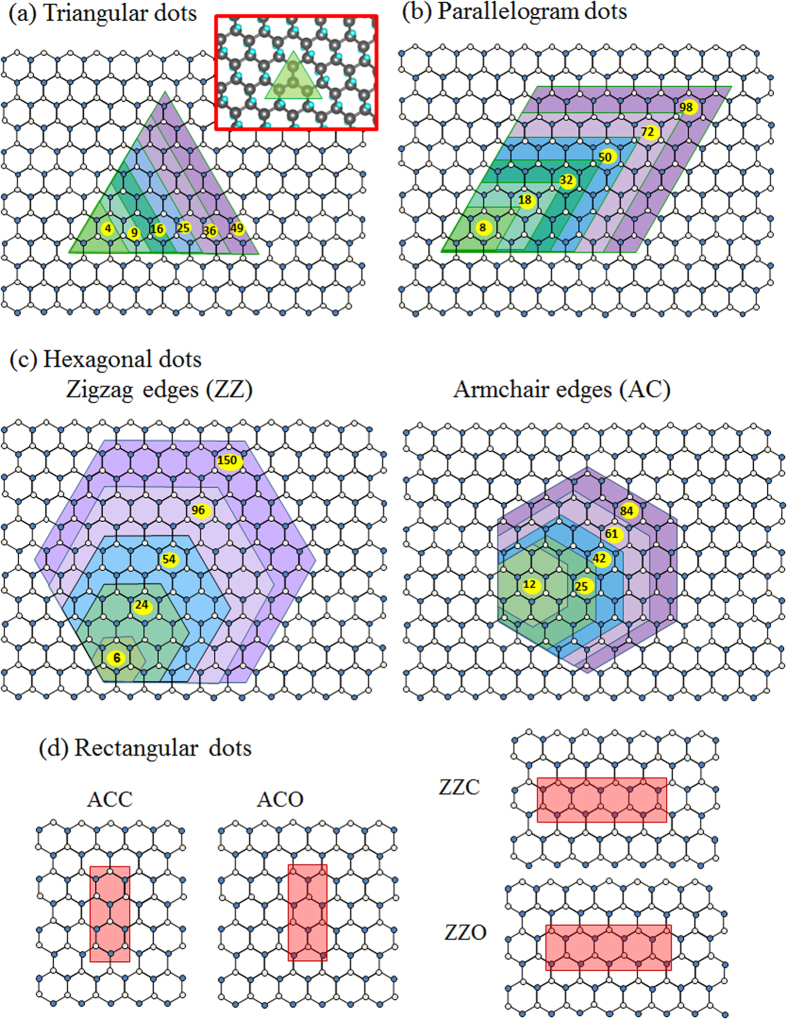
Configurations of H-vacancy clusters. (**a–c**) The triangular, parallelogram, zigzag-edged and armchair-edged hexagonal H-vacancy dots are each built from the 4, 8, 6, and 12 C clusters and expanded to contain more C atoms. (**d**) Configuration of rectangular H-vacancy clusters. Blue and white circles represent C atoms belonging to two different sublattices. Inset of (**a**) is the complete configuration of a (green-shaded) triangular H-vacancy dot consisting of 4 C atoms (in black circles), with H atoms, some hidden behind C atoms, shown in light blue circles.

**Figure 2:**
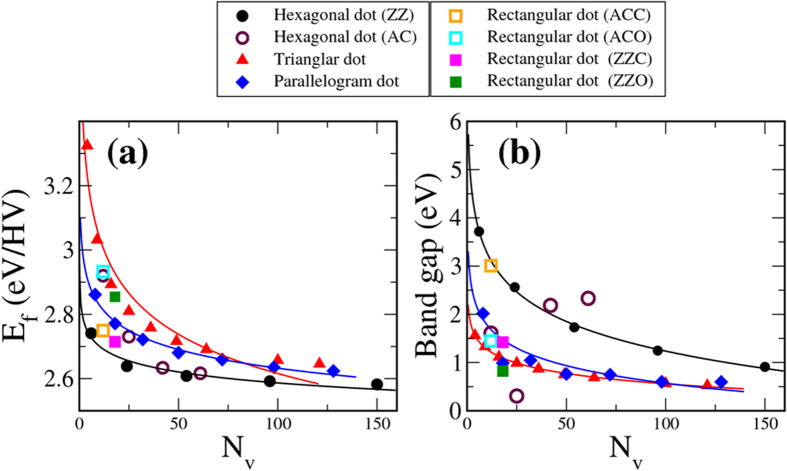
Formation energies and band gaps of H-vacancy clusters. (**a**) Formation energy of a vacancy cluster generally falls with the enlargement of its size. (**b**) Band gap of a vacancy cluster shrinks as the cluster expands. Armchair-edged hexagonal dots are the exception.

**Figure 3:**
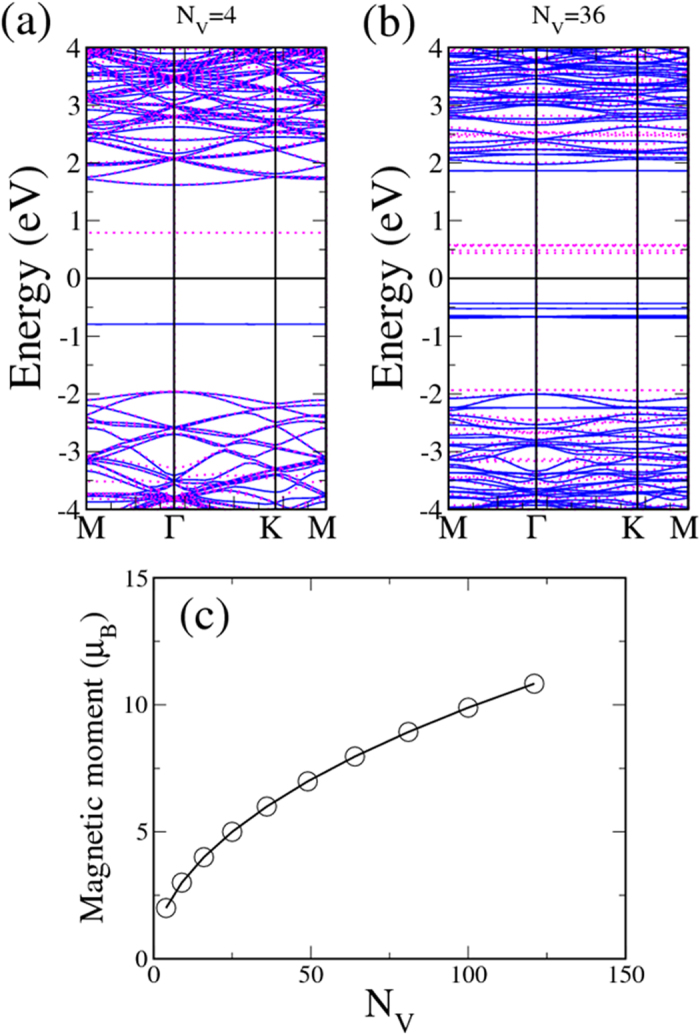
Energy bands and magnetic moments of triangular H-vacancy clusters. (**a**) Energy bands along symmetry directions for an H-vacancy cluster of 4 C atoms. Blue (red) curves are for the majority (minority) spin. (**b**) Same as (**a**) for a 36-C cluster. (**c**) Relationship between magnetic moment and number of C atoms in the vacancy cluster.

**Figure 4:**
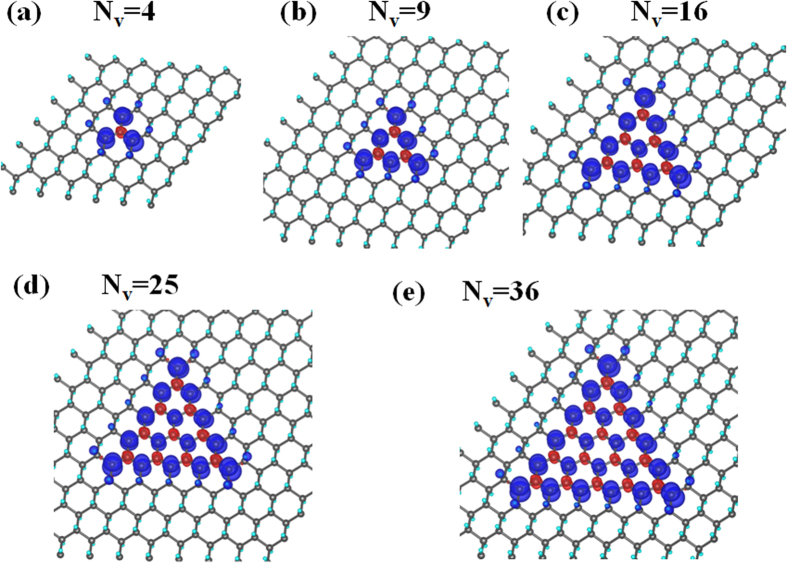
Spin densities of triangular H-vacancy clusters. (**a–e**) Distribution of the majority spin (blue) and minority spin (red) density within each of the five triangular vacancy dots.

**Figure 5:**
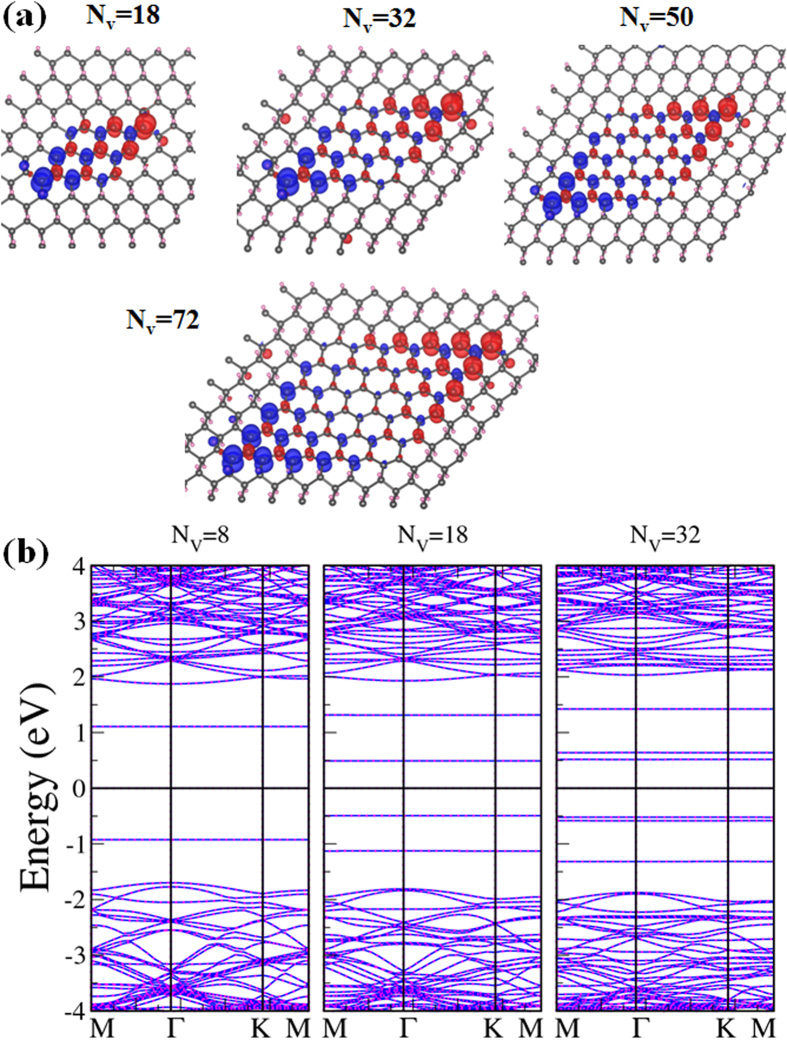
Spin densities and energy bands of parallelogram H-vacancy clusters. (**a**) Distribution of the majority spin (blue) and minority spin (red) density within each of the four parallelogram dots. (**b**) Energy bands of the three parallelogram H-vacancy dots, containing 8 (left), 18 (middle), and 32 (right) C atoms respectively.

**Figure 6:**
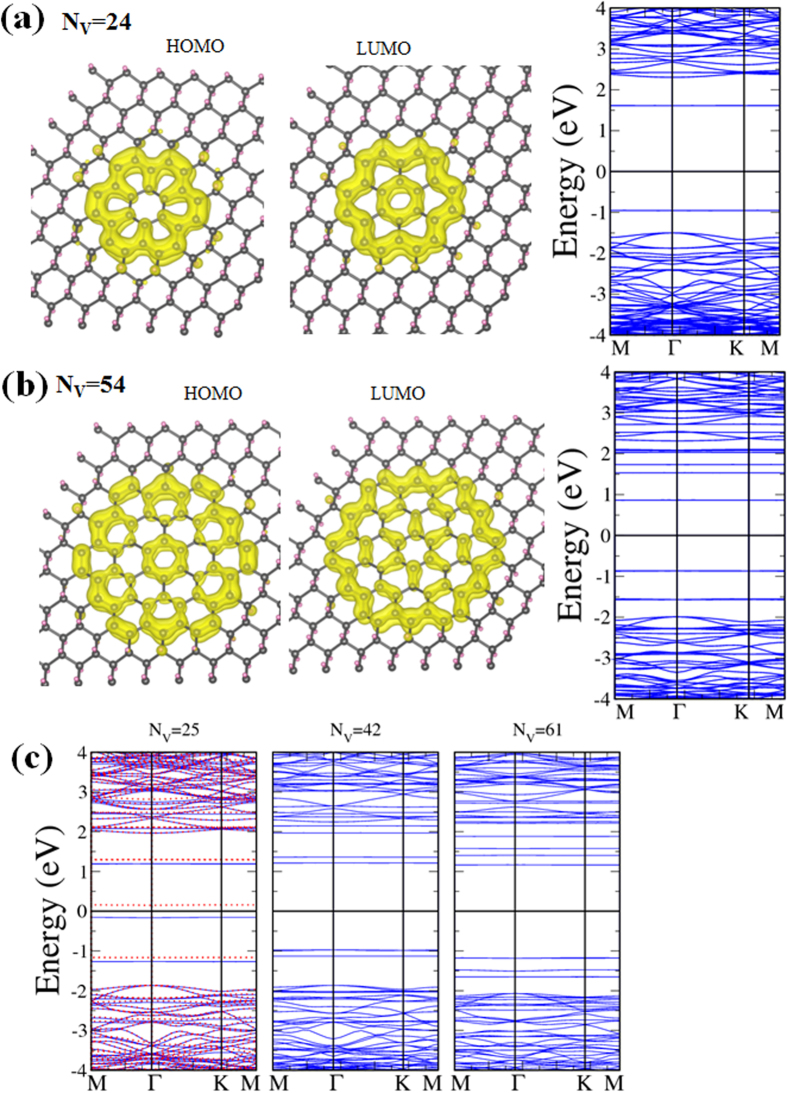
Hexagonal H-vacancy clusters. (**a**) Charge densities of HOMO and LUMO and energy bands for a hexagonal dot of 24 C atoms. (**b**) Same as (**a**) for a 54-C dot. (**c**) Energy bands for armchair-edged hexagonal dots containing 25 (left), 42 (middle), and 61 (right) C atoms respectively.

**Figure 7:**
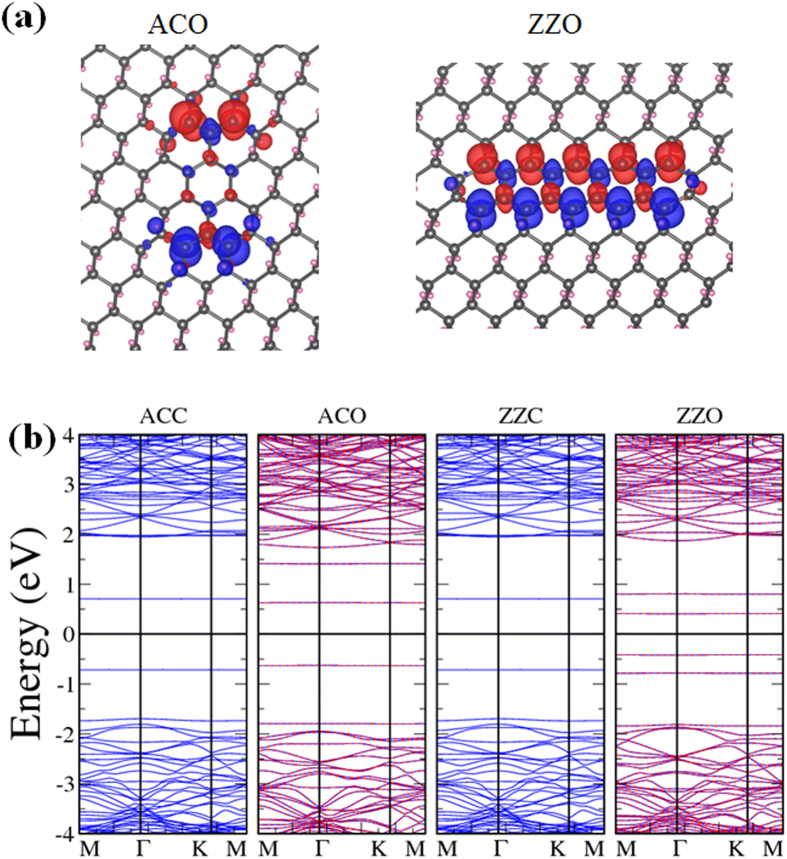
Rectangular H-vacancy clusters. (**a**) Spin densities of rectangular H-vacancy clusters ACO and ZZO. Both are antiferromagnetic with local magnetic moments. (**b**) Energy bands for ACC, ACO, ZZC, and ZZO, from left to right.
