# Supplementary material for: Electronic Structures of Clusters of Hydrogen Vacancies on Graphene
Source: Sci Rep. 2015 Oct 15;5:15310. doi: 10.1038/srep15310 (PMC4606795; doi:10.1038/srep15310)
Supplement: Supplementary Information [file srep15310-s1.pdf]

Supplementary Information for: Electronic structures of clusters of hydrogen vacancies on graphene

Bi-Ru Wu[1] and Chih-Kai Yang[2*]

[1]Department of Natural science, Center for General Education, Chang Gung University, Kueishan 333, Taiwan, ROC, [2]Graduate Institute of Applied Physics, National Chengchi University, Taipei 11605, Taiwan, ROC.

I. Differences between calculations with and without spin polarization

Each of the H-vacancy clusters has been calculated for its electronic structure with and without spin polarization. Those associated with magnetism have significant differences in their total energies under the two conditions. The energy difference ΔE is expressed as $\Delta E = E_{tot}(sp) - E_{tot}(nm)$, where $E_{tot}(sp)$ represents the total energy (per unit cell) of the vacancy dot from spin-polarized calculation and $E_{tot}(nm)$ the total energy from non-magnetic calculation. It is clear from the table below that spin-polarized results are more stable energetically and are thus presented in the article.

Triangle dots		Parallelogram dots		Rectangular dots	
N_v	ΔE (eV/cell)	N_v	ΔE (eV/cell)	Type	ΔE (eV/cell)
4	-0.54907	8	-0.00816	ACO	-0.3957
9	-0.70485	18	-0.00914	ZZO	-0.0605
16	-0.77117	32	-0.09207		
25	-0.8472	50	-0.3502		
36	-0.9153	72	-0.4698	Hexagonal dots	
49	-0.9687	98	-0.565	N_v	ΔE (eV/cell)
64	-1.0209	128	-0.6605	25	-0.01342
81	-1.0997				
100	-1.1284				
121	-1.0617				

II. Alternate definition of formation energy for H-vacancy clusters

Formation energy of an H-vacancy cluster can be defined with respect to either a free H atom or molecule. Since a free H molecule is energetically more favorable than two free H atoms, the calculated formation energy with respect to an H molecule, shown in Figure (a) below, is also different from that with respect to a free H atom in Figure (b).

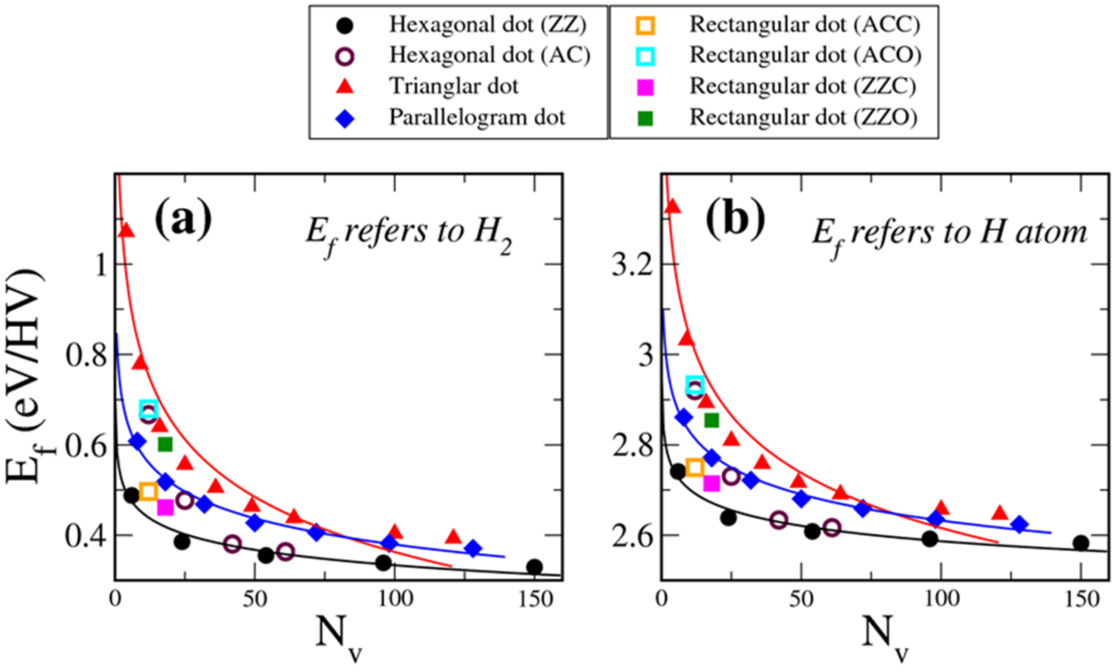
Hexagonal dot (ZZ)
Hexagonal dot (AC)
Trianglar dot
Parallelogram dot
Rectangular dot (ACC)
Rectangular dot (ACO)
Rectangular dot (ZZC)
Rectangular dot (ZZO)
(a)
E_f refers to H_2
(b)
E_f refers to H atom
E_f (eV/HV)
N_v
0
50
100
150
0.4
0.6
0.8
1
2.6
2.8
3
3.2
